# Prevalence of Urinary Tract Infection and Antimicrobial Susceptibility among Diabetic Patients with Controlled and Uncontrolled Glycemia in Kuwait

**DOI:** 10.1155/2016/6573215

**Published:** 2015-12-30

**Authors:** May Sewify, Shinu Nair, Samia Warsame, Mohamed Murad, Asma Alhubail, Kazem Behbehani, Faisal Al-Refaei, Ali Tiss

**Affiliations:** ^1^Clinical Services Department, Dasman Diabetes Institute, P.O. Box 1180, 15462 Kuwait, Kuwait; ^2^Biochemistry & Molecular Biology Unit, Dasman Diabetes Institute, P.O. Box 1180, 15462 Kuwait, Kuwait

## Abstract

Diabetic patients have higher risk of urinary tract infection (UTI). In the present study, we investigated the impact of glycemic control in diabetic patients on UTI prevalence, type of strains, and their antimicrobial drugs susceptibility. This study was conducted on urine samples from 722 adult diabetic patients from which 252 (35%) samples were positive for uropathogens. Most UTI cases occurred in the uncontrolled glycemic group (197 patients) versus 55 patients with controlled glycemia. Higher glycemic levels were measured in uncontrolled glycemia group (HbA1c = 8.3 ± 1.5 and 5.4 ± 0.4, resp., *P* < 0.0001). Females showed much higher prevalence of UTI than males in both glycemic groups (88.5% and 11.5%, resp., *P* < 0.0001). In the uncontrolled glycemia group 90.9% of the UTI cases happened at ages above 40 years and a clear correlation was obtained between patient age ranges and number of UTI cases (*r* = 0.94; *P* = 0.017), whereas in the group with controlled glycemia no trend was observed.* Escherichia coli* was the predominant uropathogen followed by* Klebsiella pneumoniae *and they were together involved in 76.2% of UTI cases. Those species were similarly present in both diabetic groups and displayed comparable antibiotic resistance pattern. These results highlight the importance of controlling glycemia in diabetic patients to reduce the UTI regardless of age and gender.

## 1. Introduction

Urinary tract infections (UTIs) are one of the most common microbial diseases encountered in medical practice affecting people of all ages [1]. Worldwide, UTIs' prevalence was estimated to be around 150 million persons per year [2].

Diabetic patients have a higher incidence of UTI than their nondiabetic counterparts [3, 4] with a higher severity UTI which can be a cause of complications, ranging from dysuria (pain or burning sensation during urination) to organ damage and sometimes even death due to complicated UTI (pyelonephritis) [5]. In 2012, the direct medical costs associated with managing UTIs in the 22 million diabetic patients in USA were estimated to be more than $2.3 billion [6]. Moreover, diabetic patients encounter further urinary urgency and incontinence during night, a condition often manifested by painful urination and retention of urine in the bladder [7]. Furthermore, those patients frequently suffer from bacterial cystitis with higher prevalence in diabetic women including higher prevalence of both asymptomatic bacteriuria and symptomatic UTI added to recurrent complications as compared to healthy women [8, 9]. In women, premenopausal and postmenopausal periods aside with sexual activity are considered increased risk factors for developing UTI [3, 8, 10]. Finally, diabetic women have up to four times more UTI risk when they are in oral treatment or insulin injection [10].

Potential explanation of the increased UTI in diabetic patients might be the nerve damage caused by high blood glucose levels, affecting the ability of the bladder to sense the presence of urine and thus allowing urine to stay for a long time in the bladder and increasing infection probability [8, 11]. Another explanation is that high glucose levels in urine improve the growth of the bacteria in the urine [12]. Additionally, the reduced blood circulation due to prolonged diabetes mellitus may result in abnormalities of the host defense system as reflected, for example, by the decrease in certain cytokines such as IL-6 and other proinflammatory cytokines in the urine of diabetic patients [13] which may increase the risk of developing infection.

Despite the fact that* E. coli* is the most frequent bacterium in UTI, other aggressive pathogens are highly prevalent in diabetic UTIs such as fungal infections,* Klebsiella*, Gram-negative rods,* enterococci*, group B* streptococci*,* Pseudomonas*, and* Proteus mirabilis* [14, 15].

Therefore, improved control of glycemia in diabetics may help in controlling the UTIs. Accurate screening for UTI in diabetic patients is also critical to enable the appropriate treatment, avoiding related complications. Nevertheless, only scarce data are available with respect to prevalence, recurrence, and microbiological features of UTI in diabetic patients with good glycemic control as compared to those with poor glycemic control.

In this study we aimed to assess the prevalence of UTI in diabetic patients referred to our specialized center, Dasman Diabetes Institute, as well as the type of microbiologically confirmed UTI and pattern of the antimicrobial drugs susceptibility in relation to diabetes mellitus in patients with good and patients with poor glycemic control.

## 2. Material and Methods

### 2.1. Study Population

The present study was carried out between April 2011 and March 2014 at Dasman Diabetes Institute (DDI), a specialized outpatient center to help diabetic patients in controlling blood glucose levels and treating their diabetes complications. The study included 252 patients with positive UTI (see Table 1 for details) out of a total number of 722 analyzed samples. Information on patient age, gender, and history of urinary frequency was obtained from the DDI Laboratory Information System (LIS). The access and use of the anonymized data analysis from the LIS for the purpose of publication were approved by the Ethical Review Board of DDI and carried out in line with the ethical guideline of Declaration of Helsinki.

### 2.2. Urine Collection and Processing

Clean voided midstream urine samples were collected in sterile special urine collection cups with the assistance of trained laboratory staff at DDI. Before sample collection, each patient was provided with a brochure and instructions explaining how to collect a correct midstream urine sample to avoid contamination. All urine samples were inoculated using a calibrated inoculation needle with 10 *μ*L of urine and each sample was inoculated on three types of media: blood agar, MacConkey agar plates, and CLED agar (Oxoid, Basingstoke, UK). All plates were incubated at 37°C for 24–48 hours for visible growth.

### 2.3. Identification of Isolated Microorganisms

Urine samples showing a colony count more than 10^4^ cfu/mL were considered to be positive for UTI. UTI isolates were identified following standard biochemical tests. Results were not considered for more than two clinical isolates obtained from the same patient and the sample was considered to be contaminated. No-growth plates were considered as sterile.

For positive urine cultures, identifications were done using automated system Microscan (Walkaway 40 SI, Siemens Healthcare Diagnostics, Sacramento, CA). Panels used for Gram-negative bacteria (NC34 and NC53) and for Gram-positive bacteria (PC21) were obtained from Siemens Healthcare Diagnostics (Sacramento, CA). For confirmation, further biochemical tests were done for both Gram-positive and Gram-negative isolates (API E20, API strep, and API staph) supplied by bioMérieux, (Durham, NC, USA).

QC strains (*Escherichia coli* ATCC 25922,* Klebsiella pneumoniae* ATCC 13883, and* Candida albicans* ATCC 10231) were supplied by American Type Culture Collection (ATCC) (Manassas, VA).

### 2.4. Susceptibility Testing

Susceptibilities of the common isolated bacteria (*E. coli*,* Enterococcus faecalis*,* Klebsiella pneumoniae*,* Serratia marcescens*,* Pseudomonas aeruginosa*,* Staphylococcus saprophyticus*,* Staphylococcus aureus*, and* Proteus mirabilis*) to selected antimicrobial agents causing UTI were examined. Antimicrobial sensitivity testing of all isolates was performed on diagnostic sensitivity test plates according to the Kirby-Bauer method [16] following the definition of the Committee of Clinical Laboratory International Standards (CLIS, 2014). Bacterial inoculums were prepared by suspending the freshly grown bacteria in 5 mL sterile saline. A sterile cotton swab was used to streak the surface of Mueller Hinton agar plates. Filter paper disks containing a designated concentration of the antimicrobial drugs obtained from Becton and Dickinson Company (Franklin Lakes, NJ) were used.

### 2.5. Statistical Analysis

Data were analyzed using the statistical software SPSS for Windows, version 17.0 (SPSS, Chicago, IL, USA). Nonparametric Mann-Whitney test was used to determine significance of difference in means between the UTI groups. Correlations between variables were calculated with Spearman's rank correlation test. *P* < 0.05 was considered to be statistically significant.

## 3. Results

### 3.1. Study Population

This study was conducted on urine samples from 722 diabetic patients received at DDI during the period between April 2011 and March 2014. Among the 722 analyzed samples, 323 (45%) were showing sterile urine samples, while 147 (20%) showed mixed growth of bacteria possibly due to improper collection of the sample. The remaining 252 (35%) samples were positive for uropathogens with colony count higher than 10^4^ CFU/mL of urine and were included in the current study analysis. The studied population was classified according to the glycemic status; patients with controlled glycemia (HbA1c < 6.5) and patients with uncontrolled glycemia (HbA1c ≥ 6.5) and their main characteristics are summarized in Table 1. The number of subjects with UTI was clearly higher in the uncontrolled glycemic group (*n* = 197, 78.2%) in comparison to the controlled glycemic group (*n* = 55, 21.8%). The mean age was significantly lower for the diabetic patients with controlled glycemia (48 ± 16 years) when compared to that (63 ± 16 years) for the diabetic patients with uncontrolled glycemia (*P* < 0.01). As expected, significantly different levels of glycemia were measured between both groups (HbA1c = 5.4 ± 0.4 and HbA1c = 8.3 ± 1.5, resp., *P* < 0.0001). Nevertheless, no clear difference was observed in the distribution of the type of diabetes, its duration, or the used treatment between the two groups (Table 1).

### 3.2. UTI and Etiology of Isolates

As summarized in Table 2, females showed much higher prevalence of UTI than males as 223 (88.5%) of UTIs of the total study population were in females versus only 29 (11.5%) in males (*P* < 0.0001). Interestingly, this gender distribution pattern was very similar in both patient groups with controlled and uncontrolled glycemia.

**Figure 1:**
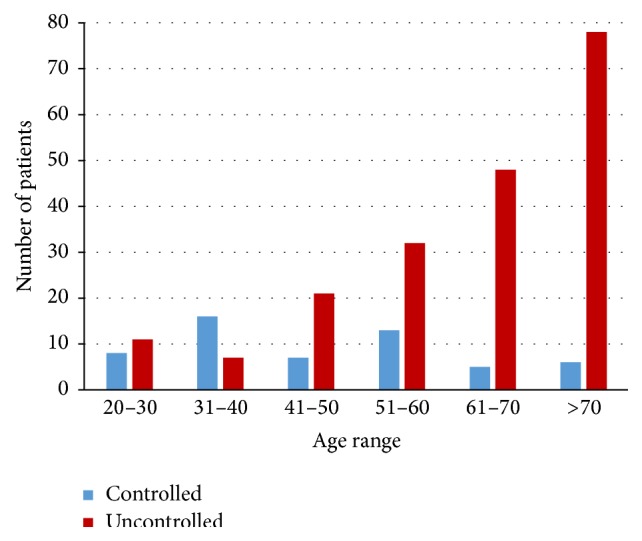
Distribution of UTI cases according to age ranges and glycemic status of our diabetic patients.

It is worth noting that, in contrast with the controlled glycemia group, in the patients with uncontrolled glycemia there is a clear increase of UTI cases with age as 90.1% of UTI cases were observed in women with an age above 40 years (Table 2). This same trend was also observed for both males and females, despite the low number of males with UTI in our study, in particular in the controlled glycemic group. Further analysis using Spearman's correlation ranking of the distribution of UTI according to the age ranges has shown a clear increase with age of UTI cases in the uncontrolled glycemia group (*r* = 0.94; *P* = 0.017) as compared to the controlled glycemia group where there was no trend of UTI cases according to age (Figure 1).

The prevalence and the distribution of Gram-negative and Gram-positive bacteria and yeast isolated from the clinical samples are shown in Table 3 for both controlled and uncontrolled glycemia groups. These isolates from both females and males represented clinically significant pathogens. As shown in Table 3,* E. coli* was the predominant pathogen isolated from urine samples in both females and males, as well as from both patient groups including 6 ESBL positive cases, all from uncontrolled glycemia patients. Indeed,* E. coli* was isolated from 57% of UTI cases in females and 37% of UTI cases in males. In our patients,* E. coli* was similarly present in both controlled and uncontrolled glycemia groups (53.3% and 58.1%, resp.) as shown in Table 3.

**Figure 2:**
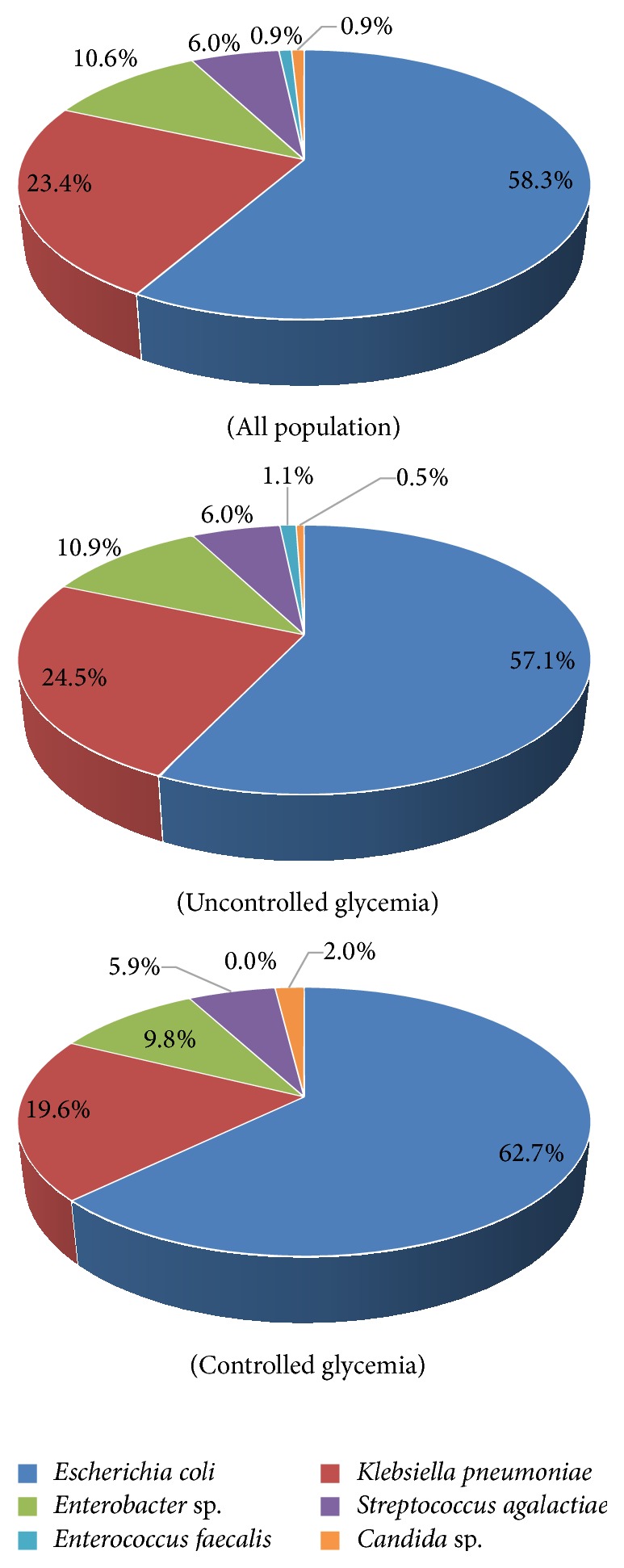
Distribution of most UTI prevalent pathogens in our study population groups.

The strain* K. pneumoniae* (21.8% of all cases) showed only 1 ESBL positive case also isolated from uncontrolled glycemia patients.* Enterobacter* species represented 10.5% of the isolated pathogens, whereas Gram-positive* S. agalactiae* (group B* streptococci*) were found in about 5.5% of the UTI cases and only about 1% of cases were assigned to yeast* Candida *species. Together,* E. coli* and* K. pneumoniae* strains are the most prevalent uropathogens and represent 76.2% of UTI cases (Table 3). In more detailed analysis and when taking into account only the 6 major strains identified in our study, the same trends in species distribution were obtained as shown in Figure 2.

### 3.3. Antimicrobial Susceptibility Pattern

**Figure 3:**
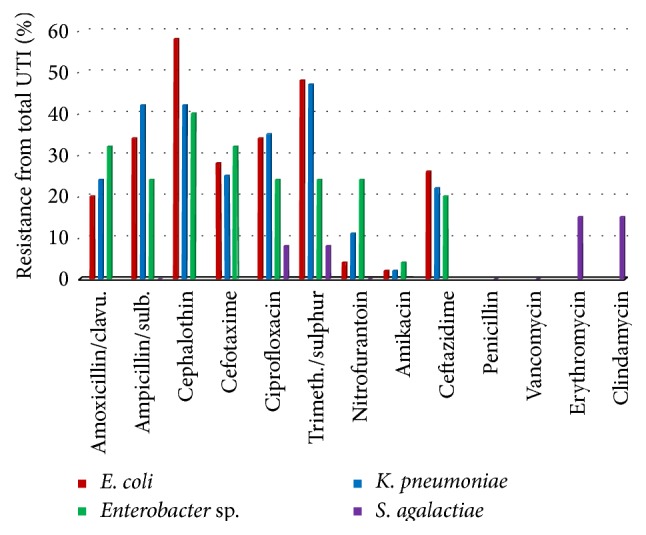
Resistance pattern for most UTI prevalent Gram-negative and Gram-positive pathogens in all population study.

The resistance pattern of UTI isolates from diabetic patients at DDI was analyzed and the results of antibiotic resistance of the most prevalent Gram-negative and Gram-positive pathogens are shown in Figure 3. Due to the limited number of diabetic patients with controlled glycemia, we did not analyze separately the resistance pattern of UTI isolates in this group. Gram-negative pathogens showed a comparable susceptibility pattern to most of the antibiotics, whereas the Gram-positive* Staphylococcus agalactiae* displayed completely different patterns (Figure 3). Indeed, the most prevalent pathogen,* E. coli*, displayed relatively high antimicrobial resistance rates against most of the tested antibiotics, that is, cephalothin (58%), trimethoprim-sulfamethoxazole (48%), ciprofloxacin and ampicillin/sulbactam (34%), cefotaxime (28%), ceftazidime (26%), amoxicillin/clavulanate (20%), nitrofurantoin (4%), and amikacin (2%). Likewise,* Klebsiella pneumoniae* showed similar patterns of resistance to trimethoprim-sulfamethoxazole (47%), ampicillin/sulbactam (42%), cephalothin (42%), ciprofloxacin (34%), cefotaxime (25%), amoxicillin/clavulanate (24%), ceftazidime (22%), nitrofurantoin (11%), and amikacin (2%), respectively.* Enterobacter *species resistance pattern was as follows: cephalothin (40%), amoxicillin/clavulanate (32%), cefotaxime (32%), ciprofloxacin (24%), ampicillin/sulbactam (24%), and ceftazidime (20%), and only 4% of strains were resistant to amikacin. These species were however more resistant to nitrofurantoin (24%) and less resistant to trimethoprim-sulfamethoxazole (24%) when compared to* E. coli* and* K. pneumoniae*. Regarding* Streptococcus agalactiae*, from the 13 isolates only 2 were found to be resistant to clindamycin and erythromycin (15%). Among those 2 isolates, one was also resistant to ciprofloxacin (8%) and trimethoprim/sulphur (8%).

## 4. Discussion 

Diabetic patients have higher risk of UTI particularly in women. In the present study, we investigated the possible impact of the glycemic control on the UTI prevalence, type of strains, and their antimicrobial drugs susceptibility in diabetic patients. Our main findings are the following: (1) there is much higher prevalence of UTI in diabetic patients with uncontrolled glycemia, (2) the glycemic control does affect the distribution of UTI according to age, but it does not affect its distribution according to gender, and (3) the etiology of the isolated strains and their antibiotic resistance pattern do not differ between patients with controlled and uncontrolled glycemia.

Analysis of our results showed that, among the 722 diabetic patients received at DDI, 35% were positive for uropathogens. This prevalence is apparently higher than the 20–30% commonly reported in diabetic patients [4, 15, 17–19]. This might be due to the fact that most of our subjects were diabetic for long periods (>10 years). Indeed, in diabetic patients, specific risk factors for UTI are usually the duration of diabetes and the presence of long-term complications, such as neuropathy, rather than current glucose control [8, 20]. It is worth mentioning that in our study there was no preselection of enrolled subjects according to gender, age, or glycemic status. Furthermore, most of the UTI cases in our study (78.2%) were found in the diabetic patients with uncontrolled glycemia in agreement with previous reports stating this trend when comparing diabetic patients (supposed to have uncontrolled glycemia) and nondiabetic subjects with normal glycemia.

Moreover, our results also showed that the majority of UTIs occurred in women (88.5%), in agreement with previous studies [19] and thereby confirming that adult women have a higher rate of UTI prevalence than men also in the diabetic population. Interestingly, this gender distribution pattern was very similar in both diabetic groups with controlled and uncontrolled glycemia suggesting that there is no impact of glycemic control on the distribution of UTI according to gender (Table 2). Similar conclusions were reported in previous studies where no significant differences were observed in the prevalence of bacteriuria both in males and in females when comparing diabetic and nondiabetic adult subjects [7, 15]. In contrast, and still comparing nondiabetic with diabetic women subjects, Geerlings et al. have reported that bacteriuria was more widespread in diabetic women with uncontrolled glycemia, [9]. As most of the previous studies on UTI in diabetic patients were carried out in women, there is limited evidence describing aspects of UTIs in diabetic men [21], and, to the best of our knowledge, the present data is the first to compare the effect of glycemic control on UTIs in males.

Despite the fact that a precise cause-effect relationship has not yet been established, multiple factors are suggested to be involved in the high occurrence of UTIs in diabetes patients. These include but are not limited to glucosuria [8], increased bacterial adherence to uroepithelial cells due to hyperglycemia [22], and neurogenic bladder [23]. In this context, Canagliflozin and Dapagliflozin, new antihyperglycemia molecules inhibiting renal glucose reabsorption and thus increasing glucosuria, were recently tested in clinical trials and were found to be associated with only a slight increase of UTI in T2D [12, 24]. This suggests that the contribution of glucosuria is limited in UTI and it does not explain its increased prevalence in diabetic patients. Nevertheless, there was a higher correlation between glucosuria and genital infection in Dapagliflozin-treated patients probably due to a greater effect of glucosuria in promoting the growth of fungal pathogens associated with genital infection as compared to bacterial pathogens typically associated with UTI [25]. In a new report, James and Hijaz have reviewed recent publications on lower urinary tract symptoms (LUTS) and UTI in diabetic women and have concluded that aging and obesity are significantly associated with worsened LUTS [26]. Glucosuria was also found to be associated with UTI and diabetic patients appeared to be at a higher risk for colonization with the virulent, extended-spectrum *β*-lactamase-producing* E. coli* and* Klebsiella* species in UTI [26]. In our studied population, obesity might be considered as a cofounder in the correlation between glycemic control and UTI as obesity rates are about 50% in Kuwait [27]. Unfortunately, we do not dispose of this parameter in our subjects to further investigate this potential hypothesis.

Age is a well-known risk factor for bacteriuria in nondiabetic females. Advanced age has been widely accepted as a risk factor for patients with type 2 diabetes mellitus. In our study at DDI, we confirmed that diabetes is associated with a higher risk of acute symptomatic UTI in postmenopausal women than younger women. Indeed, in our results, there was a clear correlation between age and UTI in the group with uncontrolled glycemia and most of the UTI cases occurred at older age (Figure 1). In contrast, in the controlled glycemia group, an almost equal distribution of the UTI cases was observed throughout all age ranges (Table 2) regardless of the gender, despite the limited number of patients in this group. This significant difference in the age of the two groups as well as the correlation with UTI might be explained by the fact that most of the patients with controlled glycemia are younger and thus more adhering to their treatment and healthy lifestyle as compared to old patients. The fact that 9% of patients with controlled glycemia have type 1 diabetes is not enough to explain this difference in trends with age despite knowing that type 2 diabetes prevalence and its complications are increased with age. It is worth noting that most of patients included in our study had diabetes for long periods (at least 10 years).

In the present study,* E. coli* was the predominant pathogen isolated from urine samples followed by* Klebsiella* in both females and males. Those species were similarly present in both groups with controlled and uncontrolled glycemia and hence were together involved in 76.2% of UTI cases. Comparable results were previously reported and confirm the predominance of those species in diabetic patients and in nondiabetic subjects [7, 15, 19, 28]. Those UTI etiological agents are also in line with previous data from the Kuwaiti general population previously reported [19, 29]. Furthermore, all the 6 ESBL* E. coli* identified in our study were isolated in diabetics with uncontrolled glycemia, in agreement with previous studies reporting higher prevalence of ESBL-producing* E. coli* and* Klebsiella* species in diabetic patients as compared to nondiabetics [15, 30]. Together, these observations may suggest a direct link between glycemic control and UTI with ESBL-producing strains. It is noteworthy that* Streptococcus agalactiae* represented around 6% of the UTI in our study in both diabetic patients with controlled glycemia and those with uncontrolled glycemia. Interestingly, and despite comparable prevalence already reported in general population analysis from Kuwait [19, 29], those numbers seem to be higher than what was reported in other populations where* S. agalactiae* was totally absent [15]. Al Benwan et al. have suggested that high prevalence of obesity and diabetes might explain this “Kuwaiti” specificity as diabetic patients are known to be predisposed to infection with this strain [19].

Our study also aimed to determine the resistance pattern for first-line antibiotics which are used at DDI outpatient clinics and which may help clinicians in the appropriate use of antimicrobial agents in diabetic patients. In our study we noted that Gram-negative strains including* E. coli* and* K. pneumoniae* were highly resistant (>45%) to trimethoprim-sulfamethoxazole, in agreement with previous reported studies from hospitals in Kuwait [19, 29]. Indeed, high resistance to this first-line antibiotic is of big concern to clinicians in Kuwait and other alternatives should be developed. Nevertheless, in our study, other antibiotics are displaying high sensitivity to all Gram-negative UTI isolates and these include amikacin (>96%), nitrofurantoin (75–96%), and amoxicillin/clavulanate (70–80%). Unfortunately, amikacin is potentially nephrotoxic and presents a risk of nephrotoxicity in patients with impaired renal function as well as in cases of diabetic patients [31]. Hence, the best acting antibiotics in our study were found to be nitrofurantoin, followed by amoxicillin/clavulanate and ciprofloxacin and then trimethoprim-sulfamethoxazole.

## 5. Conclusion

The significance of the study lies in the determination of common pathogens in diabetic patients with UTI in controlled and uncontrolled glycemia for the first time and the resistance pattern of antibiotics so that the clinicians get useful information regarding the use of antibiotics in diabetic patients. Further validation is hence anticipated in a larger diabetic population study. The study also gives evidence of differences in the etiological agents in Kuwait as compared to other regions which highlight the need of proper use of antibiotics in diabetic patients, particularly. The current study, however, has some limitations including the small number of diabetic patients with controlled glycemia analyzed and the lack of historical information on the non-UTI diabetic patients to allow detailed comparison between diabetic patients with and without UTI in relation to glycemic control. However, despite these limitations, we provided ample evidence that the control of glycemia in diabetics might help in reducing the occurrence of UTI in these vulnerable patients, specifically in aged subjects. We have also shown clear difference in the correlation between the UTI and age which seems to be directly affected by glycemic control. These findings add further evidence to the importance of tighter glycemic control in reducing the occurrence of UTI and most probably improving the clinical outcomes.

## Figures and Tables

**Table 1:** Characteristics of the study subjects with controlled and uncontrolled glycemia.

	Controlled glycemic group	Uncontrolled glycemic group
Number of patients	55 (21.8%)	197 (78.2%)
Mean age (years ± SD)	48 ± 16	63 ± 16
Type of diabetes		
Type 1	5 (9%)	15 (7.6%)
Type 2	50 (91%)	182 (92.4%)
Duration of diabetes (years)	17.26 ± 8.5	19.84 ± 8.67
HbA1c	5.4 ± 0.5	8.3 ± 1.5
Therapy		
Insulin (number)	29 (52.7%)	117 (59.4)
Metformin	27 (49.1%)	86 (43.7%)
Glimepiride	3 (5.5%)	7 (3.6%)
Sitagliptin	4 (7.3%)	12 (6.1%)

**Table 2:** Age and sex distribution of patients with positive UTI included in this study.

Glycemic status	Patients age groups	Gender		Total
		Male	Female	
Controlled	Average	54 ± 13	47 ± 17	48 ± 16
	20–30	2	6	8
	31–40	0	16	16
	41–50	0	7	7
	51–60	5	8	13
	61–70	0	5	5
	>70	1	5	6
	Total	**8**	**47**	**55**

Uncontrolled	Average	65 ± 12	63 ± 16	63 ± 16
	20–30	1	10	11
	31–40	0	7	7
	41–50	0	21	21
	51–60	7	25	32
	61–70	4	44	48
	>70	9	69	78
	Total	**21**	**176**	**197**

**Table 3:** Microbial uropathogens isolated from urine of our diabetic study population.

UTI pathogens	Uncontrolled glycemia		Controlled glycemia	
	Males	Females	Males	Females
Gram-negative microorganisms				
*E. coli*	6	99	5	27
*K. pneumoniae*	4	41	2	8
*K. oxytoca*	0	0	0	3
*Raoultella ornithinolytica*	0	5	0	0
*P. aeruginosa*	1	2	0	2
*P. mirabilis*	2	0	0	0
*Citrobacter koseri*	2	1	0	0
*Citrobacter* *bummannii*	0	1	0	0
*Citrobacter freundii*	0	1	0	0
*Morganella morganii*	0	1	0	0
*Kluyvera *species	0	1	0	0
*Miscellaneous GNB*	2	4	0	2

Gram-positive microorganisms				
*Staphylococcus epidermidis*	0	3	0	2
*Staphylococcus warneri*	0	2	0	0
*Staphylococcus sciuri*	2	2	0	0
*Streptococcus agalactiae*	2	9	1	2
*Enterococcus faecalis*	0	2	0	0
*Miscellaneous GPB*	0	1	0	0

Yeast				
*Candida glabrata*	0	1	0	0
*Candida albicans*	0	0	0	1

Total	21	176	8	47
